# Stem Cell Repair of the Microvascular Damage in Stroke

**DOI:** 10.3390/cells9092075

**Published:** 2020-09-11

**Authors:** Madeline Saft, Bella Gonzales-Portillo, You Jeong Park, Blaise Cozene, Nadia Sadanandan, Justin Cho, Svitlana Garbuzova-Davis, Cesar V. Borlongan

**Affiliations:** 1 University of Michigan, Ann Arbor, MI 48104, USA; saftmad@umich.edu; 2 Northwestern University, Evanston, IL 60208, USA; bellagonzales-portillo2024@u.northwestern.edu; 3 Department of Neurosurgery and Brain Repair, University of South Florida Morsani College of Medicine, Tampa, FL 33612, USA; youjeongpark@usf.edu (Y.J.P.); justincho@usf.edu (J.C.); sgarbuzo@usf.edu (S.G.-D.); 4 Tulane University, New Orleans, LA 70118, USA; bcozene@tulane.edu; 5 Georgetown University, Washington, DC 20057, USA; nas146@georgetown.edu

**Keywords:** ischemia, microvasculature, blood–brain barrier, diaschisis, bone marrow

## Abstract

Stroke is a life-threatening disease that leads to mortality, with survivors subjected to long-term disability. Microvascular damage is implicated as a key pathological feature, as well as a therapeutic target for stroke. In this review, we present evidence detailing subacute diaschisis in a focal ischemic stroke rat model with a focus on blood–brain barrier (BBB) integrity and related pathogenic processes in contralateral brain areas. Additionally, we discuss BBB competence in chronic diaschisis in a similar rat stroke model, highlighting the pathological changes in contralateral brain areas that indicate progressive morphological brain disturbances overtime after stroke onset. With diaschisis closely approximating stroke onset and progression, it stands as a treatment of interest for stroke. Indeed, the use of stem cell transplantation for the repair of microvascular damage has been investigated, demonstrating that bone marrow stem cells intravenously transplanted into rats 48 h post-stroke survive and integrate into the microvasculature. Ultrastructural analysis of transplanted stroke brains reveals that microvessels display a near-normal morphology of endothelial cells and their mitochondria. Cell-based therapeutics represent a new mechanism in BBB and microvascular repair for stroke.

## 1. Introduction

Stroke is currently the fifth leading cause of death in the US, and someone dies of one approximately every 4 min. Stroke occurs due to the interruption of blood flow to the brain, and is typed as ischemic or hemorrhagic. Ischemic stroke makes up approximately 87% of total strokes. Ischemic stroke survivors may not fully recover and develop long-term disabilities [1,2,3], such as hemiparesis, cognitive deficits and dependence in daily activities [4]. Stroke is a life-threatening disease with limited therapeutic options. Cell therapy has emerged as an experimental stroke treatment and BBB impairment is a key pathological manifestation of ischemic stroke.

Cerebral functional insufficiency in chronic stroke might be due to pathological changes in brain areas remote from the initial ischemic lesion, for example, diaschisis. In this review, the BBB is implicated in subacute diaschisis. Additionally, BBB competence in chronic diaschisis using a transient middle cerebral artery occlusion (tMCAO) rat model is discussed as well. BBB alterations can be demonstrated in not only the ipsilateral hemisphere, but also the contralateral hemisphere, an area with remote brain structures not directly affected by ischemia. The microvascular damage in the subacute phase likely reveals ischemic diaschisis, and should be considered in the development of treatment strategies for stroke. The direct targeting of cell delivery into the injured vasculature might be a feasible approach for stroke treatment, and endothelial progenitor cells (EPCs) serve as a promising cell source for BBB restoration in stroke. The ability to preserve the mitochondria and increase these organelles’ pinocytosis via cell-based therapeutics represents a new neurorestorative mechanism in BBB repair.

Microvascular damage in ischemic stroke demonstrates ischemic diaschisis. In this review, the investigation of subacute diaschisis in ischemic stroke rat models exhibits stroke-induced pathological disturbances in ipsilateral and contralateral brain areas, and causes microvascular damage with BBB breakdown in remote brain microvessels. Additionally, widespread microvascular alterations in ipsilateral and contralateral brain hemispheres suggests continued BBB damage in chronic ischemic stroke. Because of the rampant pathological microvascular changes in remote brain areas in both subacute and chronic ischemic diaschisis, such vascular damage presents as a therapeutic target for stroke. The use of bone marrow-derived mesenchymal stem cells has been initiated to treat patients with acute ischemic stroke. Here, we discuss the potential use of EPCs as an effective cell source for BBB restoration in stroke by promoting neurovascular repair and preserving the mitochondrial morphology of the microvasculature.

## 2. Acute Pathological Changes in Post-Ischemic Stroke

Following an ischemic stroke, substantial alterations in both cellular and molecular activity take place due to stroke-induced cerebral pathology [5,6]. Stroke-induced ischemia in the brain can manifest focally, globally, transiently or permanently, spurring oxygen and glucose deprivation, as well as the depletion of other vital nutrients in affected regions after stroke [7]. As time goes on, stroke pathology progresses through injurious developments in the ischemic core and penumbra [7]. After the initial ischemic stroke injury, a cerebral pathological cascade occurs and may further cause widespread neuronal damage [5,6,8]. Furthermore, formulating effective therapeutic strategies to combat post-ischemic stroke damage has proven to be an arduous task due to the convoluted and divergent nature of stroke pathology.

Stroke pathology due to vascular injury is time-dependent and can be classified into three types: acute (minutes to hours), subacute (hours to days) and chronic (days to months). The acute and subacute stages of ischemic stroke can be grouped into one period, as secondary cell death following both phases drastically overlap [5,6,8]. Notably, the BBB holds a pivotal role in stroke pathophysiology. BBB impairment post-ischemic injury is spurred by disturbances in cerebrovasculature [9,10,11,12,13]. Regarding patients with acute ischemic stroke, 12 h after ischemic stroke symptoms appeared, perfusion-CT was utilized to detect BBB functionality [14]. Following reperfusion in middle cerebral artery occlusion (MCAO) rat models, either focal, permanent or transient, BBB impairment worsened and was associated with cerebral blood flow levels [15]. In the ischemic hemisphere of mice, findings demonstrated that BBB disruption is upregulated at one hour post-reperfusion [11]. In MCAO rats, it takes between 3 and 5 h [10,16]. Importantly, a small amino acid tracer was observed traversing the BBB 6 h after the MCAO [17]. Nonetheless, extensive BBB openings have also been perceived immediately after ischemic stroke [18]. On the other hand, ischemic rat models have demonstrated delayed openings of the BBB ranging from 6 to 24 h post-forebrain ischemia [18]. Even though ischemic/reperfusion damage has been shown to spur biphasic, “open-close-open” permeability from the BBB distinguished by a refractory term [10,12,18,19], the BBB openings have also lasted between 4 and 5 weeks [20,21]. Furthermore, long-term BBB opening, engendered early in acute ischemic stroke, can exacerbate ischemic damage and lead to drastic post-stroke BBB interruption [20,21]. BBB leakiness induced by ischemia in the brain can evolve into substantial and potentially life-threatening clinical consequences, spurring cerebral edema and swelling [7,22,23]. In addition, elevated BBB leakage may exacerbate the spontaneous formation of ischemia-induced brain hemorrhages, known as hemorrhagic transformation [24]. Although there have been comprehensive examinations of BBB permeability and its role in ischemic stroke pathology, studies that go beyond the acute stage and the cerebral hemisphere, characterized by preliminary ischemic injury, are warranted in order to fully comprehend the effects of BBB leakage in stroke [7].

In-depth investigations into stroke pathology have brought about the phenomenon of diaschisis, an abrupt change in activity in regions of the brain far-off from the ischemic lesion generated by stroke-induced impairments. Transhemisphere diaschisis was first recognized through blood flow and metabolism alterations in unilateral and contralateral ischemic hemispheres in the brain [25]. After its original identification, in-depth investigations into the occurrence of diaschisis have elucidated its association with particular cerebral impairments far-off from the focal ischemic lesion [26,27,28,29]. As indicated by clinical outcomes and patient recovery, the acute, subacute and chronic stages following stroke can be distinguished by crossed cerebellar diaschisis, thalamic diaschisis and cortical diaschisis respectively [30,31,32,33]. Ischemic stroke damage in humans spurred distant variations in blood flow and/or metabolism in contralateral areas of the brain up to 14 days following the initial injury [34,35]. The consequent depletion of oxygen and nutrients engenders potential chronic damage to the brain [7]. Regarding this, on days 7 and 28 post-transient MCAO, permutations in contralateral excitability were ubiquitous in the neocortex of rats [36,37], triggering cerebral dysfunction [7]. The distant excitability induced by ischemic stroke has been deemed as transcortical diaschisis [37], occurring as a consequence of pervasive corticostriatal connectivity deterioration [7].

Nevertheless, in the context of the subacute phase, BBB functionality and the associated pathology in distal brain regions have received less focus [7]. A comprehensive examination of the subacute stage is warranted, as deficits in neurological function and recovery post-stroke bear significant correlation with BBB permeability and associated cerebrovascular impairments. In addition, the level of injury generated by acute ischemic stroke may play a role in remote post-acute circumstances [7]. Since the subacute phase fills the gap between the acute and chronic stages, it may heavily impact the extent of ischemic diaschisis in the brain, and therefore serves as a potential target for stroke therapy [7].

The effects of subacute diaschisis on BBB stability and associated pathological mechanisms in contralateral regions of the brain were evaluated in focal ischemic stroke rat models [7]. Using the tMCAO model, rats demonstrated drastic BBB mutations in the ipsilateral hemisphere 7 days post-surgery, depicted by substantial Evans Blue (EB) parenchymal extravasation, autophagosome build-up, significant impairment to neurons, demyelination, and upregulation of highly active astrocytes and microglia in the striatum, motor and somatosensory cortices [7]. Additionally, the contralateral hemisphere exhibited vascular injury as observed through ultrastructural and immunohistochemical evaluation. Notably, endothelial cells (ECs), demonstrating swelling and vacuolation and characterized by a high number of autophagosomes, pericyte deterioration and perivascular edema, were detected in the contralateral striatum and motor cortex, indicating significant alterations in the BBB. The contralateral striatum, motor and somatosensory cortices also showed drastic EB extravasation, a higher rate of endothelial autophagosome formation, unbridled astrogliosis, significant microglial activity, lower levels of myelin and pervasive neuronal pyknosis [7].

Moreover, BBB impairment in distal cerebral microvessels and endothelial autophagosome build-up could be correlated with the injuries observed in the ipsilateral and contralateral brain regions spurred by focal ischemic stroke [7]. Importantly, diaschisis induced by stroke may be associated with subacute microvascular deficits, and therefore serves as a potential target for stroke therapy.

By utilizing a focal ischemic stroke rat model, subacute diaschisis could be explored [7,26,27,28,29,36,37]. Damage predominantly manifested in the ipsilateral hemisphere, and involved BBB modifications, autophagosome accumulation, demyelination, a rise in activated microglia and reactive astrocytes, and neuronal damage. However, injury to the BBB was also found in the contralateral hemisphere. These changes included swollen and vacuolated ECs, with enlarged mitochondria and autophagosomes, pericyte degeneration, perivascular edema and EB extravasation. The findings also demonstrated augmented autophagosome formation, activated microglia, astrogliosis, decreased myelin, and neuronal pyknosis. The pathological changes observed indicate subacute diaschisis with a substantial contribution from the damaged BBB.

A major pathologic characteristic of subacute tMCAO involves BBB impairment in the ipsilateral and contralateral striatum and motor cortex [7,26,27,28,29,36,37]. Capillary EC damage and pericyte and astrocyte degeneration compromise BBB integrity. A ruptured microaneurysm in the ipsilateral motor cortex was discovered, indicating vascular damage. This damage was likely associated with the presence of thrombogenic cells, harbingers of blood clots, and spontaneous hemorrhagic transformation. Indeed, hemorrhagic transformation is a severe complication of ischemic stroke [37]. Mechanisms of hemorrhagic transformation in ischemic stroke have been distinguished [38]. For instance, a loss of basal lamina components is associated with microvascular permeability and can likely lead to hemorrhagic effects [39]. Ultrastructural abnormalities in capillary EC in the hemisphere contralateral to tMCAO were also discovered. These injuries may signal BBB damage and vascular changes in the subacute phase of ischemia. Additionally, utilizing 3T MRI, the relationship between BBB permeability and edema formation after ischemic injury was displayed [40]. Results demonstrated edema formation in the contralateral striatum 4 h post-reperfusion. Further research is warranted to investigate BBB integrity in cerebral diaschisis during different ischemic stages.

### 2.1. Parenchymal Extravasation

Parenchymal extravasation plays a role in secondary cell death following an ischemic stroke, and can be linked to diaschisis and BBB impairment. An in vivo study illustrated that EB extravasation was amplified in the MCAO group when compared to the control group, and could be associated with exacerbated BBB integrity [41]. A high level of extravasated EB was discovered in the ipsilateral hemisphere in comparison with the control, reflecting vascular leakage at the ischemic injury site [10,42,43,44]. An increased level of EB extravasation was also observed in the contralateral hemisphere. Interestingly, neovascular permeability is a possible cause of the increased level of EB extravasation [10,42,43,44]. In vitro, higher levels of microvascular compartments derived from parenchymal compartments were correlated with augmented EB extravasations, which could be linked to BBB breakdown in vitro under OGD/R conditions [45]. Another preclinical study demonstrated that tMCAO-induced BBB dysfunction was correlated with drastic EB extravasation in the contralateral striatum, motor and somatosensory cortices [7]. Subacute diaschisis in a focal ischemic stroke rat model, which displayed substantial BBB damage, manifested in EB extravasation, indicating the link between parenchymal extravasation, BBB leakage and the development of subacute diaschisis. Endothelial transcellular permeability should be reviewed due to the presence of damaged capillary ECs in both ipsilateral and contralateral hemispheres [46]. The BBB was open 7 days post-tMCAO, and previous studies indicate BBB leakage for up to several weeks [20,21]. However, this vascular leakage appeared to be limited to the brain.

### 2.2. Autophagosome Accumulation

Autophagy in capillary ECs following ischemic stroke may heavily contribute to cell impairment and BBB alteration. It is still unknown whether enhanced autophagy is part of cell survival or death in an ischemic environment. Autophagosome accumulated in ipsilateral and contralateral capillary ECs following tMCAO. Autophagy plays a pivotal role in managing cell homeostasis by the degradation of cytosolic components via an autophagosomal–lysosomal pathway [47]. Excessive autophagy may degrade critical cell components and induce cell death [48]. It is still unknown whether enhanced autophagy is part of cell survival or death in an ischemic environment. The initiation of autophagy has the possible ability to protect neurons from ischemic insult and progress towards neuronal necrosis by building molecular blocks [49,50]. Notably, autophagosomes are critical for EC survival in early-stage post-ischemia. EC autophagy potentially contributes to cell impairment and BBB alteration. Further examination needs to be conducted to explore these mechanisms in subacute diaschisis. It may be essential to explore metabolic levels in remote brain areas due to findings revealing metabolic disturbances in non-ischemic brain regions post-MCAO in rats [51].

### 2.3. Microglial and Astrocyte Changes

Additionally, reactive astrocytes and activated microglia populated in remote striatum, motor and somatosensory cortices 7 days post-tMCAO, potentially indicating an inflammatory response. Inflammation is a major event in ischemic stroke [52,53,54]. Inducing these glial cells plays a key role in the enhancement of neuronal damage through the secretion of pro-inflammatory cytokines and chemokines. Inflammation also collaborates with other factors, generating oxidative stress and endothelial matrix in ischemic vascular endothelial injury [55,56], which increases BBB permeability [57]. Reactive astrogliosis in the subventricular zone (SVZ), disrupted neuroblast migratory scaffolds and ependymal cell alteration were discovered in the ischemic lesion site in a mouse tMCAO model [58]. Stroke induces changes in the SVZ neurogenic compartment. Blood–CSF barrier impairment may also potentially contribute to the glial response in the contralateral hemisphere.

### 2.4. Neuronal Pyknosis

Neuronal pyknosis was also found in subacute ischemia in remote striatum, motor and somatosensory cortices. This post-ischemia damage potentially contributes to stroke pathology and inhibits recovery processes. Secondary neuronal damage and glial cell reactions were explored via two ischemic rat models [59]. The results displayed various damaged neurons within the ipsilateral ventroposterior and reticular thalamic nuclei post-tMCAO. Suture tMCAO, which develops edema, may lead to secondary damage in the thalamic nuclei. EC damage and perivascular edema were viewed at the ultrastructural level in both ipsilateral and contralateral hemispheres after tMCAO. This may induce further neuronal damage, followed by glial cell activation [59].

### 2.5. Demyelination

In the ipsilateral hemisphere, diminished myelin was found in brain structures with neuronal damage. Decreased myelin was found in the contralateral hemisphere with reduced striatosome size. Communication between different regions of the brain is vital to normal brain function, making white matter damage harmful [6,60]. In order to comprehend damage to white matter tracts, it is essential to understand the involvement of oligodendrocytes in myelin production at the subacute ischemic phase. Contralateral brain injury mediated through subacute diaschisis is possibly caused by initially acute ischemic brain areas via transneuronal pathways. Contralateral brain pathology is dependent on the severity of acute ischemic damage. BBB damage may also indicate pathological vascular changes associated with neurodegenerative processes. This can contribute to the advancement of cognitive decline and post-stroke dementia. Around 30% of patients who experienced a stroke developed post-stroke dementia within the span of two years [61]. One of the major risk factors in post-stroke patients includes vascular damage, which is dependent on the severity of the cerebral insult and white matter injury [62]. There is still much controversy over how stroke may induce neurodegenerative dementia [63,64,65]. To conclude, subacute diaschisis accompanied a focal ischemic stroke rat model. The breakdown of the BBB and endothelial autophagosome accumulation in brain capillaries was noted. Microvascular damage in the subacute phase is linked with ischemic diaschisis, indicating that the repair of injured vasculature proximal and distal to the primary ischemic brain site should be examined when establishing potential treatment approaches for stroke. One viable therapeutic approach involves repairing the damaged BBB. This would inhibit the exacerbated degeneration of surviving neurons. Furthermore, the BBB serves as a significant therapeutic target that can be used to establish neuroprotective strategies in stroke.

## 3. Chronic Pathological Changes in Post-Ischemic Stroke

The magnitude of the primary stroke lesion is important in predicting patient outcomes. However, the location of the lesion is also essential, especially during chronic stages. Motor recovery and functional outcome in hemiplegic stroke patients during the chronic stage had a stronger correlation with brain lesion size and location compared to that of only lesion size [66]. Post-stroke recovery may also be dependent on functional alterations in remote brain structures, which are located far from the primary ischemic lesion. Specifically, transhemispheric diaschisis can be determined by observing the changes in blood flow and metabolism in the hemisphere contralateral to unilateral cerebral ischemia [25,34]. Recovered stroke patients 6 months after severe hemiparesis displayed similar lesion and recovery networks in the thalamus and occipital cortex [30]. MCAO models of transient ischemia further reveal transcortical diaschisis in the neocortex [36,37]. This suggests a possible extension of corticostriatal connection degeneration, which was demonstrated in focal cortical rat stroke models in cortical regions, an affected brain region distant from the primary location [67]. Determining the competence of BBB may be critical in developing effective stroke therapeutic strategies. Studies have targeted BBB disruption post-stroke to understand the complex pathological changes [68,69,70,71,72]. Both acute ischemic stroke patients and MCAO rat models have demonstrated increased BBB permeability [14,15,16,17]. BBB leakage separated by a period of ischemic-reperfusion lesion was observed between 3 and 24 h after MCAO [10,12,18,19]. However, some studies revealed BBB leakage lasting up to 5 weeks, potentially causing further post-stroke brain injury [20,21].

### 3.1. Endothelial Cell amd Pericyte Impairment

As noted in the preceding section, BBB pathological changes occur in both the ipsilateral hemisphere and contralateral brain regions, indicating the presence of subacute ischemic diaschisis [7]. An association of microvascular damage with BBB alterations is detected in remote brain areas from initial lesions during the subacute ischemic stage, suggesting that BBB competence and diaschisis may evolve in chronic ischemia. Indeed, consistent BBB damage in the ipsilateral striatum and motor cortex of rats occurred at 30 days after tMCAO, characterized by endothelial and pericyte cell impairment [73,74]. Such BBB alterations extended to the contralateral hemisphere, again accompanied by vacuolated ECs and degenerated pericytes. These manifested pathological changes were observed in both primary and remote injury areas, supporting the occurrence of BBB damage in chronic ischemia.

### 3.2. Parenchymal Extravasation

Chronic parenchymal extravasation was also revealed via electron microscopy imaging in ipsilateral and contralateral hemispheres [73,74]. The quantitative analysis of extravasated EB in brain parenchyma provides evidence of BBB abnormalities leading to vascular leakage. EB levels in both hemispheres were significantly higher compared to those of non-ischemic controls. Additionally, the EB extravasation level in the contralateral hemisphere was significantly less than the EB level in the ipsilateral hemisphere. Compared to a previous study where significant Evans Blue (EB) levels in both the ipsilateral and contralateral hemispheres in subacute tMCAO setting were demonstrated [7], roughly half of EB extravasations were noted in the chronic stage. There was a considerable reduction in leakage from subacute to chronic stage. However, significant capillary leakage after tMCAO (30 days) supports findings from previous studies regarding BBB leakage [20,21], indicating pervasive BBB damage during chronic stages that may lead to perivascular edema. The possible emergence of EB extravasation due to the impairment of EC in both hemispheres can also be explored. EB extravasation may be induced by decreasing the integrity of tight junctions in hypoxic conditions, demonstrated in in vitro [75,76] and ex vivo [77]. studies. Additionally, an elevated EB level in brain parenchyma may be due to neovascular permeability, which was evident in the occurrence of angiogenesis and vessel proliferation shortly after ischemic insult in MCAO models and stroke patients [78,79,80,81]. Because leakage occurs in new cerebral vessels until a fully functional BBB forms [82], despite vascular remodeling in neurological post-stroke recovery [83], vascular leakage is potentially due to failed ED solidity and neovascular permeability, allowing harmful neuronal substances to enter the central nervous system (CNS) and contributing to secondary brain damage. tMCAO in rats presented significant neurological and cognitive dysfunction after 1 month, in addition to tremendous neuronal cell losses in ipsilateral striatum post-stroke compared to the contralateral striatum [83,84]. Studies have also found correlations between lesion size and pathological changes of the brain in tMCAO rats 21 to 60 days after stroke [85,86,87], and a significant decrease in apical dendritic spine density of neurons in the peri-infarct cortex in aged mice 5 months post-focal cerebral cortical ischemia [88]. These studies examined pathological neuronal changes in the brain regions of the initial lesion. However, ultrastructural analysis discovered substantial amounts of neurons with swollen endoplasmic reticulum, or edematous cytoplasm and demyelinated axons, in the striatum and motor cortex of both hemispheres post-chronic ischemia. More studies regarding the condition of neurons in brain areas remote from the initial ischemic injury in chronic ischemia settings are needed to bolster our understanding of BBB competence in chronic ischemia.

### 3.3. Autophagosome Accumulation

Excessive autophagosome production in both ipsilateral and contralateral striatum and motor cortex areas was observed in previous studies, primarily in subacute post-tMCAO settings, which was also exhibited in ECs in chronic stages due to the upregulation of Belcin-1 expression [73,74]. Increased autophagosomes within penumbra neurons induced further neuronal damage after the initial ischemic injury [89,90,91], highlighting the importance of autophagy in cell degradation through the autophagosomal–lysosomal pathway. Capillary analyses of the striatum and motor cortex of both hemispheres displayed Beclin-1 overexpression in ECs, especially in the ventral striatum of ipsilateral and contralateral hemispheres. Because the ventral striatum neighbors a vital area for afferent brain connections, called the interstitial nucleus of the posterior limb of the anterior commissure (IPAC) [90,91,92], affected capillaries may harm amygdala–striatal afferent connections. Understanding BBB integrity in the connected remote brain areas may be a key factor in post-stroke diaschisis because the amygdala–striatal transition area connects to the striatum through cortico- and thalamostriate pathways, in addition to involvements in projections to substantia nigra [91,93,94]. Additionally, there was significant Beclin-1 overexpression of ipsilateral M2 and M1 motor cortex areas in EC capillaries, and a higher expression of Beclin-1 was observed in M1 compared to M2. Beclin-1 expression in the contralateral capillary did not significantly differ from controls. However, elevated expression was observed in the capillary endothelium of the motor cortex. The evident capillary alterations resulting from autophagosome accumulation may induce neuronal injury in post-chronic ischemia by potentially causing vascular leakage. Studies have demonstrated progressive neuronal damage post-degeneration of corticofugal axonal fibers extending into the contralateral hemisphere and descending along tracts in brain structures, such as the cerebral cortex, thalamus, brainstem and spinal cord [95,96,97,98,99]. Predicting functional recovery depends on corticospinal tract disruption in chronic stroke patients, indicating that increasing Beclin-1 expression correlates with a reduction in endothelia and a rupturing of the lumen of the EC membrane due to excessive autophagosome accumulation 30 days after tMCAO. Additionally, BBB dysfunction and leakage are potentially caused by uncontrolled autophagosome accumulations, which was determined by quantitative analysis of EB extravasation. This large buildup of autophagosomes in EC indicates severely damaged, post-ischemic stress cells. Although EC initiates self-repairing mechanisms, many EC fail to fully recover through autophagocytosis, and eventually die, contributing to capillary leakage. It is possible that the initial stroke may have shifted the EC to an anaerobic metabolism, while failing to switch back to aerobic metabolism in chronic settings post-stroke [100]. Therefore, the observed EC leakage may be due to a malfunction of normal transport mechanisms and cellular metabolism.

### 3.4. Astrocyte and Microglial Changes

The analysis of vascular damage, such as BBB impairment, in cerebral brain structures represents a key pathological feature of chronic tMCAO at the ultrastructural levels [73,74]. Abnormal microvascular activities and astrocyte degeneration insult the strongly supported BBB integrity in chronic post-stroke settings. Major BBB leakage was evident in the striatum and motor cortex capillaries of the ipsilateral hemisphere, while significant but less severe BBB alterations were observed in the same brain structures in the hemisphere contralateral to tMCAO after 30 days [73,74]. This emergence of neuronal damage in remote brain regions opposite to the initial brain lesion may indicate a potential correlation between structural vascular changes and chronic diaschisis.

Astrocyte reactivity in both ipsilateral and contralateral hemispheres displayed significant increases in glial fibrillary acid protein (GFAP) immunoreactivity in striatal areas of the ipsilateral hemisphere, while significant increases in GFAP expression were observed in the dorsal area of the contralateral striatum despite enhanced astrocyte reactivity in the striatum regions. The overexpression of bilateral astrocyte was only present in the dorsal striatum, but the causation of this phenomenon is unknown. Astrogliosis in the striatum may indicate spatial differences in astrocyte reactivity due to the structural location of the striatal area relative to the corpus callosum. Studies have demonstrated the degeneration of transcallosal fibers in subacute and chronic stroke settings using MRI [99]. To explore this possibility, further research should be conducted at different post-stroke stages in in vivo human models. No significant differences in GFAP immune response in motor cortices between tMCAO and controls were present in both hemispheres. However, the expression of GFAP in the M2 ipsilateral hemisphere was significantly higher than that in the contralateral hemisphere, indicating reactive astrogliosis of the striatum in the ipsilateral side and possible post-ischemic inflammation. Inflammation is known to make a major contribution to stroke pathology [52,54]. Neuroinflammation may be correlated to active astrocytes due to the activation of pro-inflammatory cytokines and chemokines. An unusual location of microglia in the striatum and motor cortex was discovered through ultrastructural examinations, indicating the migration of microglia to the capillary wall and suggesting a possible induction of vascular endothelial inflammation. Another study analyzed astrocyte degeneration in the striatum 21 days after an hour of MCAO in rat models [101]. The study did not demonstrate astrocyte degeneration in the contralateral striatum or cortex post-ischemia. However, astrocyte degeneration was evident in the capillaries and striatum and motor cortex neurons of both hemispheres 30 days after tMCAO. Moreover, the immunohistochemistry of both hemispheres revealed a separation of astrocyte from capillary lumen. Microvascular astrocyte contacts led to cerebral edema in rat models with permanent MCAO [102], which progressed over time during the acute stages of focal ischemia. Symptoms were also observed, including astrocyte swelling, focal detachment from basement membrane, and basement membrane degradation. The impairment of astrocytes post-ischemia during subacute and chronic stages is likely due to perivascular edema formation [7], which may be associated with fluid balance in the cerebral cortex. Stable cooperation between the capillary endothelium and astrocytes is necessary to maintain a healthy BBB, and could be a possible target for stroke therapy by reviving normal BBB functions [103,104].

Investigation of damaged BBB integrity in the cerebral hemisphere capillaries (ipsilateral and contralateral to the initial stroke lesion) in chronic post-ischemic rats may reveal chronic diaschisis. Large autophagosome accumulation and astrocyte degeneration led to EC impairment, ultimately inducing extensive microvascular damage and neuronal degeneration in chronic ischemia. Chronic diaschisis should be included in stroke therapeutic strategies, primarily reviving endothelial and astrocytic integrity for BBB repair. Cell therapy is a possible stroke treatment option for BBB repair by replacing dysfunctional ECs through EPC transplantation. Combining cell therapy with pro-inflammatory inhibitors may prove to be a worthy stroke treatment.

## 4. Stem Cell Repair of Microvascular Damage

Currently, the only FDA-approved treatment for ischemic stroke is tissue plasminogen activator (tPA) for dissolving the blood clot and improving blood flow in the brain. When tPA is administered intravenously up to 4.5 h after stroke onset in patients with acute ischemic stroke, it reduces mortality and increases the rates of independent ambulation when thrombolytic treatment is given early [105]. However, tPA is only available for acute ischemic stroke within a limited time frame, and treatment options are limited. Cell therapy that targets the subacute and chronic phase of acute stroke serves as a promising treatment [106].

In MCAO-induced rat models, various cell types including bone marrow stromal cells [107], umbilical cord blood cells [108] and mesenchymal stem cells [109] have proven beneficial in increasing neurotrophic growth factors in the ischemic tissue, reducing apoptosis in the penumbral lesion zone, reducing ischemic damage and restoring cerebral blood flow [109,110,111,112,113]. With regard to the different stem cells, bone marrow stromal cells have demonstrated the ability to increase vascular endothelial growth factor (VEGF) expression and promote angiogenesis after stroke [107]. While umbilical cord blood cells have been shown to improve functional recovery in stroke rat models, limited research has investigated their use in human models [108]. Additionally, mesenchymal stem cells have also been explored and can be easily obtained. However, the safety and efficacy of this type of cell therapy has still not been determined and depends on the mode of cell administration [109]. Based on this information, the use of bone marrow-derived mesenchymal stem cells [114,115] or mononuclear cells [116,117] was initiated for the treatment of acute ischemic stroke in patients, and positive outcomes were achieved, but the efficacy of the proposed treatments remain inconclusive [118]. Further research is needed to determine more effective stem cell-based therapies for ischemic stroke treatment.

### 4.1. BBB Repair in MCAO-Induced Rodent Models

A feasible approach for stroke treatment may involve directly targeting cell delivery into the injured vasculature. Since pervasive BBB impairment is an important factor in ischemic stroke pathogenesis, BBB repair might serve as a primary target for cell therapy development. An early study has shown that the intrastriatal transplantation of bone marrow stromal cells in MCAO rats restored local cerebral blood flow and decreased BBB permeability [110]. Additionally, another study showed decreased BBB leakage and increased tight junction protein (occludin) expression in the ischemic border of cell-treated rats [107]. However, EPCs might be a more effective cell source for BBB restoration due to their vascular phenotype. The intravenous administration of human-cord-blood-derived EPCs into rats 24 h post-MCAO promoted angiogenesis and neurovascular repair, and also reduced ischemic infarct volume and improved neurobehavioral outcomes [119]. While the mechanism of action underlying the therapeutic benefits of cell therapy in stroke is still unclear, the effects of transplanted EPCs on mitochondria and pinocytosis can be examined, and grafted EPCs may be feasible in preserving mitochondrial morphology and promoting pinocytic activity. By evaluating the effects of intravenously transplanted human bone marrow EPCs into MCAO rats using electron microscopy, the functionality of stem cell-mediated BBB repair and ultrastructure components can be revealed with a specific focus on determining capillary endothelium integrity in brain areas remote from the initial ischemic insult.

The ability of transplanted human bone marrow EPCs (hBMEPCs) to repair the BBB in adult Sprague-Dawley rats subjected to tMCAO can be evaluated via electron microscopy [120]. When β-galactosidase hBMEPCs were intravenously transplanted 48 h post-tMCAO, stroke rats displayed widespread vascular repair in the bilateral striatum and motor cortex and robust cell engraftment within capillaries. hBMEPCs-transplanted stroke rats exhibited a near-perfect morphology of ECs, pericytes and astrocytes, and the morphology of the mitochondria was normal in ECs and perivascular astrocytes as well. Numerous pinocytic vesicles with engrafted cells were also observed in the transplanted stroke rats, and the robust engraftment and functionally of the hBMEPCs helped to get rid of stroke-altered vasculature. Therefore, in cell-based therapies, preserving the mitochondria and augmenting pinocytosis is imperative in BBB repair for stroke.

With intravenously transplanted β-gal pre-labeled hBMEPCs into rats 48 h after tMCAO, the rats experienced vascular repair in the brain structures of both hemispheres via transplanted cell engraftment into the vascular wall, which helped to re-establish BBB integrity post-stroke [120]. BBB repair in cell-treated tMCAO rats can be supported by the extensive vascular engraftment of β-gal pre-labeled hBMEPCs, normal morphology of ECs, healthy pericytes, myelinated axons, the astrocyte processes surrounding vessels, increases in mitochondria with normal morphology and decreases in abnormal mitochondria, lack of perivascular edema, and numerous pinocytic vesicles at the abluminal and luminal membranes with more vesicles in the ipsilateral than the contralateral hemisphere. hBMEPCs can be successfully incorporated and engrafted into the vascular wall in both the ipsilateral and contralateral remote brain areas, and leads to the high functionality of engrafted cells. Intravenously transplanted hBMEPCs contribute to BBB repair after stroke by being able to successfully engraft into the cerebrovasculature and exert pinocytic activity [120]. Since BBB repair was observed in the early stages of stroke, additional studies are required to examine long-term post-transplant effects.

At 7 days post-tMCAO, BBB damage in the ipsilateral and contralateral striatum and motor cortex was observed [120], consistent with previous results demonstrating subacute diaschisis in tMCAO rats [7]. In hBMEPC-treated tMCAO rats, stroke-induced BBB pathologies, such as capillary EC damage, pericyte and astrocyte degeneration and perivascular edema, were undetected as compared to untreated rats. Additionally, the engraftment of transplanted β-gal pre-labeled hBMEPCs was confirmed in numerous cerebral capillaries in both brain hemispheres by electron microscopy after HRP-DAB staining. Thus, a single HRP-DAB staining of β-gal pre-labeled hBMEPCs provides sufficient EM analysis in vivo [120].

### 4.2. Astrocytes and Microglia Analyses

Intravenously transplanted hBMEPCs in tMCAO rats provide beneficial effects that are visualized by quantitative mitochondrial morphology analysis within ECs and perivascular astrocytes. Mitochondrial alterations can lead to cellular energy deficits and oxidative stresses that result in cell death, and mitochondrial dysfunction can occur after stroke which results in the reduced number and size of the mitochondria in astrocytes [121], neuronal mitochondrial swelling and fragmentation [122], excitotoxic calcium entry overload [123] and deficient astrocytic support to neuronal functions [124]. Following an ischemic stroke, significantly fewer mitochondria with normal morphology are present in perivascular astrocytes, not only in the ipsilateral, but also in the contralateral striatum and motor cortex. Potentially, dysfunctional mitochondria can cause astrocyte end-feet degeneration, which worsens post-ischemic BBB damage. Undertsanding the role of mitochondria in post-stroke EC degeneration and categorizing their abnormal morphologies might lead to the use of mitochondrial condition as a potential biomarker of BBB alterations after stroke, and in determining treatment effects. Moreover, targeting the mitochondria might serve as a potential therapeutic approach for stroke [125]. The intravenous transplantation of hBMEPCs into tMCAO rats significantly decreased abnormal mitochondria and resulted in increased numbers of mitochondria with normal morphology within ECs and perivascular astrocytes [120]. However, the role of mitochondrial dysfunction is still unclear in terms of whether it plays a primary or secondary role in the events leading up to BBB impairment in stroke, and further studies are needed.

### 4.3. Pinocyctic Vesicles Analysis

In cell-treated post-tMCAO animals, numerous pinocytic vesicles found solely in engrafted hBMEPCs were present [120]. Typically, adult brain capillary ECs lack pinocytic vesicles [126], which causes low BBB permeability by inhibiting the transcellular passage of molecules across the barrier [127]. Although the BBB prevents many macromolecules from entering the brain, proteins and peptides might cross the cerebral endothelium, via receptor-mediated or adsorptive-mediated transcytosis involving the uptake of material into a cell by invagination of the plasma membrane and its internalization in a membrane-bound vesicle [128,129]. Pinocytosis involves the indigestion of fluid or soluble materials from the environment, and these solutes can be incorporated into small pinocytic vesicles for digestion or passage.

The pinocytic vesicles were found only in engrafted hBMEPCs within the cerebral capillaries of cell-treated post-tMCAO rats, and represent a new line of investigation for cell therapy [120]. Administered stem cells, such as EPCs derived from the human bone marrow, can undergo in vivo proliferation, differentiation, maturation, migration and adherence to stroke-damaged capillaries. After completion of this process, 5 days post-transplant, once the cells are engrafted into the vascular wall, pinocytic activity in these cells begins and indicates the high functionality of the newly introduced cells in the replacement of damaged ECs. Additionally, the absence of perivascular edema at the site of vesicles’ appearance indicates that transplanted cells use pinocytosis for draining fluid from the damaged brain capillary areas where they have been incorporated. The significantly high number of pinocytic vesicles detected in engrafted hBMEPCs in the ipsilateral vs. contralateral hemisphere in both striatum and motor cortex supports the importance of pinocytic activity in post-stroke microvascular recovery [120].

### 4.4. Limitations and Future Directions of Cell Therapy-Induced BBB Repair in Stroke

Although intravenously transplanted hBMEPCs replaced post-stroke damaged ECs, it is possible that the administered cells were involved in capillary sprouting, or might have extravasated to the brain parenchyma via paracellular migration across cell–cell junctions similarly to leukocyte diapedesis in various pathological conditions, given the new vessel formation [126,127]. Transplanted hBMEPCs might promote neovascularization and/or stimulate angiogenesis in ischemic-damaged cerebral areas, enhancing BBB integrity [120]. Since BSCB damage has also been shown in rat models of ischemic stroke [74], it is important to determine the effect of transplanted cells on the repair of this barrier in the spinal cord to confirm vascular restoration in areas remote from the initial tMCAO insult. It is also essential to define BBB permeability after cell transplantation so as to confirm the benefit of this proposed stroke treatment. The involvement of EPCs in re-reendothelialization during the neovascularization or formation of collateral vessels after ischemia has been discussed in comprehensive reviews detailing the safety and efficacy of EPCs for further clinical applications in stroke patients [130].

Additionally, transplanted hBMEPCs might not only exogenously but also endogenously enhance post-stroke vasculogenesis, via the secretion of angiogenic or growth factors. Local transplantation of the human EC cell line improved endogenous vasculogenesis and neurogenesis by the VEGF signaling pathway [131]. Significantly increased angiogenesis and neurogenesis were also seen in MCAO mice treated with murine BMEPCs, mediated via the endothelial nitric oxide synthase (eNOS)/brain-derived neurotrophic factor (BDNF) signaling pathway [132]. Future studies will address this possibility (see Table 1 and Table 2).

In conclusion, β-gal pre-labeled hBMEPCs intravenously transplanted into rats 48 h after tMCAO engrafted within the capillary wall at 5 days after its administration and closely participated in the vasculature repair of the BBB in subacute stroke [120]. The analysis of microvessels, in the bilateral striatum and motor cortex of animals with unilateral ischemic stroke, identified widespread pinocytic vesicles, which is uncommon in adult brain capillary ECs but recognized in ECs of cell-treated stroke animals. Alongside the preservation of normal mitochondrial morphology, the observation of BBB repair by newly engrafted hBMEPCs sheds new light on research and a better understanding of disease pathology and treatment. In particular, this research paves the way for the development of cell-based therapeutics directed at enhancing mitochondrial function and pinocytotic activity in stroke [120].

## 5. Conclusions

Stroke is a devastating disease, and further investigation regarding the underlying pathophysiological changes is warranted so as to unveil an effective neurodegenerative treatment. Acute and chronic microvascular damage, as a result of ischemia, may act as a potential mechanism to target when contemplating stem cell therapy [120]. Similarly, maintaining BBB function is integral to mitigate worsening symptoms post-ischemia [73]. Pathological alterations in remote areas of the brain may be linked back to both vascular damage and impaired BBB, and a novel treatment may attenuate this stroke-induced diaschisis [73]. Intravenously administered hBMEPCs have shown promising effects in murine tMCAO models [120]. BBB vasculature repair has been observed, as well as increased pinocytosis and preserved mitochondrial function. This opens a new path of research within cell-based therapeutics that leads towards elucidating a robust therapy designed to maintain BBB and microvasculature function, consequently alleviating diaschisis and providing beneficial outcomes to stroke patients.

## Figures and Tables

**Table 1:** Stroke and Microvascular Disease. This table highlights milestone discoveries highlighting the pathological links between stroke and microvascular disease.

Study	Discovery
Hamann et al., 2003 [133]	Microvascular damage manifests in the brain just three hours after intracarotid clot injection. Compared to the non-ischemic control side, a 16% decrease in microvasculature density and a 10% decrease in stained microvessels was observed after a short amount of time. Damage was also seen in areas where regional cerebral blood flow levels had returned to normal (Hamann et al., 2003).
Vosko et al., 2003 [134]	Microvascular basal lamina damage is limited to areas with apparent diffusion coefficient reduction (ADC-R) in magnetic resonance imagining (MRI) in rat thromboembolic occlusion of middle cerebral artery. ADC-R positive basal ganglia (cortical) areas exhibited decreased microvascular density and stained microvascular areas compared to the non-ischemic hemisphere (Vosko et al., 2003).
Vosko et al., 2006 [135]	Increased duration of ischemia and reperfusion exacerbate microvascular damage and infarct volume in a mouse focal cerebral ischemia and reperfusion model. Infarct volume increases directly as duration of ischemia and reperfusion increases. A sharp decrease in collagen type IV positive vessels was also observed in ischemia-afflicted areas (Vosko et al., 2006).
Liu et al., 2011 [136]	Rho kinase (ROCK) propels microvascular damage via increasing matrix metalloproteinase 9 (MMP9) in a rat middle cerebral artery occlusion model. A linear relationship between ROCK and microvascular damage progression was observed in the brain. ROCK inhibitor, fasudil, increased Laminan expression and was found to inhibit MMP9 ischemia-induced expression. Taken together, data indicate that ROCK plays a role in the manifestation of microvascular damage (Liu et al., 2011).
Moisan et al., 2014 [137]	MRI of microvasculature can provide valuable information of states of vessels and indicates post-stroke plasticity. An acute stage was observed from days 1–3 and displayed increased concentrations of angiopoietin-2 (Ang2), vascular endothelial growth factor receptor-2, and endothelial NO synthase. The transition stage, days 3–7, was characterized by the presence growth factors Ang1, TGFβ1 and tyrosine kinase with endothelial growth factor-like domains and immunoglobulin-like domains, stromal-derived factor-1 and chemokine receptor types 4 and 3. The subacute phase, days 7–25, presented with augmented Ang1, Ang2, TGFβ1, VEGF and VEGFR-1 levels. Together, these phases indicate the apparent microvascular plasticity present after stroke and bolster its potential as a target for therapy (Moisan et al., 2014).
Zhang et al., 2017 [138]	Mestastasis-associated lung adenocarcinoma transcript 1 (Malat1) provides neuroprotection in ischemic stroke. In vitro, increased levels of Malat1 were seen in culture post-oxygen glucose deprivation. Similarly, increased levels were found in cerebral microvessels in a mouse in vivo study. Inhibition of Malat1 resulted in increased cell death and expression of pro-apoptotic genes. Furthermore, Malat1 KO mice exhibited increased infarct volume, poor sensorimotor function, and low neurological scores (Zhang et al., 2017).
Koizumi et al., 2018 [139]	Transgenic mice with human ApoE4 gene, known to predispose humans to small vessel disease, display decreased neocortical cerebral blood flow in a cerebral hypoperfusion model. This is caused by lowered vascular density and impaired homeostatic mechanisms. Post-hypoxia, mice experienced damage to the white matter of the corpus callosum and aggravated cognitive impairments. These changes may be behind the hypoxic-ischemic lesions observed in the subcortical white matter of humans with ApoE4 allele (Koizumi et al., 2018).

**Table 2:** Use of Stem Cells for Stroke. This table highlights important studies on the use of stem cells for the repair of stroke-induced microvascular damage.

Study	Discovery
Shyu et al., 2006 [140]	Peripheral blood hematopoietic stem cells (CD34+) (PBSCs) were administered intracerebrally in chronic cerebral ischemia rat models. PBSCs differentiated into vascular ECs and spurred angiogenesis, improving local cortical blood circulation into the ischemic region. Importantly, macrophage and microglial cells generated by stem cells, along with the expression of beta1 integrin, may promote vascular repair in the brain (Shyu et al., 2006).
Thored et al., 2007 [141]	Ischemic stroke spurs hypoxia, vessel density alterations, and angiogenesis in the ipsilateral subventricular zone and the striatum. Following MCAO, neuroblasts migrated to injured vasculature at 2, 6 and 16 weeks, indicating the involvement of neuroblasts in promoting vascular regeneration and fortifying vessel density post-stroke. Moreover, vascular repair serves as a significant therapeutic target in post-stroke recovery (Thored et al., 2007).
Teng et al., 2008 [142]	In vitro, cerebral ECs derived from normal rats co-cultured with conditioned medium from stroke-affected neural progenitor cells spurred the development of a capillary tube. Their findings indicate that the phenomena of angiogenesis and neurogenesis post-stroke are synergistic and are potentially regulated by the vascular endothelial growth factor (Teng et al., 2008).
Yang et al., 2010 [143]	Rat and human bone marrow stromal stem cells (MSCs) were delivered to MCAO rats intravenously. Neurological recovery with both forms of MSCs was observed and could be associated with newly formed vasculature in the infarct area (Yang et al., 2010).
Wei et al., 2015 [144]	Hypoxic preconditioned BMSCs were administered intranasally to rat pup models of neonatal stroke. The BMSCs drastically alleviated BBB leakage, upregulated angiogenesis and neurogenesis, rehabilitated the cerebrovasculature, and ameliorated cerebral blood circulation into the ischemic cortex (Wei et al., 2015).
Mao et al., 2015 [145]	t-MCAO rats were transplanted with skin-derived precursor cells (SKPs) in the cortex and the striatum. SKPs generated upregulated angiogenesis and neurogenesis, indicating their potential as a rehabilitator against stroke-induced vascular damage (Mao et al., 2015).
Ryu et al., 2016 [146]	Human neural stem cells were administered to the subventricular zone of MCAO rats, displaying focal cerebral ischemia. The stem cells boosted the proliferation of ECs in the ischemic region, upregulated endogenous neural stem cell development into mature neuronal-like cells, and stimulated angiogenesis (Ryu et al., 2016).
Park et al., 2017 [147]	MCAO experimental models received either one human umbilical cord-derived mesenchymal stem cell (hUCB-MSC) dose two days after the surgery or repeated hUCB-MSC therapy (two and nine days post-surgery). Both groups demonstrated higher rates of neurogenesis and angiogenesis, suggesting these cells’ therapeutic potential in acute ischemic stroke (Park et al., 2017).
Huang et al., 2017 [148]	Human umbilical cord blood (hUCB) mononuclear cells (MNCs) were delivered intraarterially to rat stroke models. The HUCB MNCs drastically improved cerebral blood flow to the afflicted sector during the hyperacute stage of stroke. In addition, cerebrovascular reactivity and density were upregulated. Notably, a number of the engrafted hUCB MNCs evolved into ECs and interacted with ECs belonging to the host, indicating crosstalk between grafted cells and blood vessels (Huang et al., 2017).
Yang et al., 2018 [149]	After OGD, ADSC-derived exosomes (ADSC-Exos) enhanced the migratory capabilities of brain microvascular ECs (BMECs) and augmented angiogenesis through the miR-181b-5p/TRPM7 pathway (Yang et al., 2018).
